# Circulating levels of testosterone, 17 beta-oestradiol, luteinising hormone and prolactin in postmenopausal breast cancer patients.

**DOI:** 10.1038/bjc.1983.35

**Published:** 1983-02

**Authors:** G. Secreto, C. Recchione, A. Cavalleri, M. Miraglia, V. Dati

## Abstract

Serum testosterone, 17 beta-oestradiol, luteinising hormone (LH) and prolactin were measured in 28 postmenopausal breast cancer patients after mastectomy and in 30 postmenopausal normal controls. In the patient group, mean levels of oestradiol, LH and prolactin did not differ significantly from those of the control group. Mean testosterone levels were higher in breast cancer patients than in normal controls, either considering the overall groups (P less than 0.001) or dividing them into subgroups according to years since menopause. Breast cancer patients were divided into 2 subgroups according to time since mastectomy: 19 patients had been examined within a year of mastectomy and 9 patients some years after mastectomy. Testosterone, but not oestradiol, LH or prolactin values in each subgroup were still significantly higher (P less than 0.001 and P less than 0.02, respectively) than in normal controls. Years since menopause were significantly correlated with testosterone (r = 0.533, P less than 0.01) but not with the other hormones in the cancer group. These results confirm our previous findings of increased urinary testosterone values in postmenopausal breast cancer patients and support the hypothesis that androgens may play a role in the development of breast cancer.


*Br. J. Cancer* (1983), **47**, 269–275

### Circulating levels of testosterone, 17β-oestradiol, luteinising hormone and prolactin in postmenopausal breast cancer patients

G. Secreto, C. Recchione, A. Cavalleri, M. Miraglia & V. Dati

*Hormone Research Laboratory, Istituto Nazionale per lo Studio e la Cura dei Tumori, Milan, Italy*

**Summary** Serum testosterone, 17β-oestradiol, luteinising hormone (LH) and prolactin were measured in 28 postmenopausal breast cancer patients after mastectomy and in 30 postmenopausal normal controls. In the patient group, mean levels of oestradiol, LH and prolactin did not differ significantly from those of the control group. Mean testosterone levels were higher in breast cancer patients than in normal controls, either considering the overall groups ($P<0.001$) or dividing them into subgroups according to years since menopause. Breast cancer patients were divided into 2 subgroups according to time since mastectomy: 19 patients had been examined within a year of mastectomy and 9 patients some years after mastectomy. Testosterone, but not oestradiol, LH or prolactin values in each subgroup were still significantly higher ($P<0.001$ and $P<0.02$, respectively) than in normal controls. Years since menopause were significantly correlated with testosterone ($r=0.533$, $P<0.01$) but not with the other hormones in the cancer group. These results confirm our previous findings of increased urinary testosterone values in postmenopausal breast cancer patients and support the hypothesis that androgens may play a role in the development of breast cancer.

Sex hormones and possibly prolactin may play an important role in the control of neoplastic growth of breast cancer, but results of hormonal determination have often been contradictory. Most of the studies have found no significant increase in the circulating levels of oestrogens and prolactin (Nagasawa, 1979; Nisker & Siiteri, 1981; Zumoff, 1981) in breast cancer patients, and the few reports of testosterone levels are conflicting, normal values having been found by some authors (Bird *et al.*, 1981; Borkowski *et al.*, 1977; Horn & Gordan, 1974) and increased levels by others (Adami *et al.*, 1979; McFadyen *et al.*, 1976). Malarkey *et al.* (1977) reported increased testosterone levels in premenopausal and normal testosterone levels in postmenopausal patients.

In the present work we report circulating levels of testosterone, oestradiol, prolactin, and luteinising hormone (LH) in postmenopausal breast cancer patients and in normal controls. The finding of increased testosterone levels in breast cancer patients supports our previous findings based on urine measurements (Grattarola *et al.*, 1974, 1975).

### Patients and methods

### *Breast cancer patients*

Twenty-eight patients who had previously undergone mastectomy for infiltrating carcinoma of the breast were studied. All of them were disease-free at the time of hormonal examination. The age of these patients was 47–77 y (mean 58.5 y) and all had been postmenopausal for 1–28 y. The patients were divided into 3 subgroups according to years since menopause: 10 patients were 1–5 y postmenopausal, 8 were 6–10 y postmenopausal, and 10 were >10 y postmenopausal.

Twenty-five of the 28 patients married between the age of 21 and 36 years and had had at least one full-term pregnancy between the ages of 23 and 41 years. Three patients were single and had had no pregnancy.

The patients were divided into 2 subgroups according to the interval between mastectomy and hormonal examination: 19 patients were examined from 2–12 mo since mastectomy and 9 patients were examined from 2–17 y since mastectomy.

No patient had been previously ovariectomized and none had been treated with hormones or chemotherapy for breast cancer. None of them had been taking any hormonal drug for at least 6 mo before testing or digitalis, antihypertensive or psychotropic drugs for chronic disease.

### *Control subjects*

Thirty normal controls selected from healthy women fulfilled the following criteria: (a) no abnormality of the breast on clinical examination; (b) no benign or malignant neoplasm elsewhere; (c) no endocrine, metabolic or chronic disease; (d) no family history of breast cancer; (e) no involuntary

Correspondence to: G. Secreto, Hormone Research Laboratory, Istituto Nazionale Tumori, Via Venezian 1, 20133 Milano, Italy.

Received 8 June 1982; accepted 21 October 1982.

0007-0920/83/020269-07 $02.00

© The Macmillan Press Ltd., 1983

infertility; (f) no menstrual cycle irregularities in the past.

Ages were 49–77 y (mean 57.4 y), and all had been postmenopausal for 1–30 y. They were divided into 3 subgroups according to years since menopause: 12 were 1–5 y postmenopausal, 11 were 6–10 y postmenopausal, and 7 were >10 y postmenopausal.

Twenty-four had married between the ages of 18 and 30 y and delivered at least one full-term newborn between the ages of 20 and 32 y. Six control subjects were single and had had no pregnancy.

Table I reports the ranges and means for the various characteristics of the patients and the controls.

*Methods*

Hormonal determinations were performed on peripheral blood samples drawn between 9 a.m. and 11 a.m. Blood was allowed to clot at room temperature, centrifuged and serum separated and stored at −20°C until assayed. Testosterone, oestradiol, LH and prolactin levels were determined by radioimmunoassay in the same blood samples.

For the determination of testosterone, preliminary chromatography was performed with a Sephadex LH-20 column according to the method of Collins *et al.* (1972). However, since we wished to eliminate dihydrotestosterone we performed the assay of testosterone only on the fraction of elution between 0.9 and 2.0 ml immediately after the azobenzene.

The cross-reaction of the antibody towards testosterone-3-oximebovine serum albumin, which we used, was <0.1% for all the principal steroids, except for dihydrotestosterone (17β-hydroxy-5α-androstan-2-one), 5-androsten-3β-17β-diol, and 5α-androstan-3α-17β-diol. These steroids were thus eliminated by chromatography.

The separation of free testosterone from that bound to the antibody was performed by use of the double antibody method (Ismail *et al.*, 1972). The levels of testosterone found by interpolation of the standard curve were corrected for the recoveries (which were from 50–70%), whereas no correction was made for the blanks, since these were negligible.

The levels of oestradiol were determined without preliminary chromatography (Ricci & Sacconi, 1976), using a rabbit antibody raised against 17β-oestradiol-6-carboxymethyloxime-bovine serum albumin, which was highly specific (Ricci & Sacconi, 1976), and polyethylene glycol for separating bound from free steroid (Desbuquois & Aurbach, 1971). LH and prolactin were respectively determined with the methods of Midgley (1966) and Sinha *et al.* (1973). LH was expressed in international milliunits of the Second International Reference Preparation of Human Menopausal Gonadotropin (2nd IRP-HMG); prolactin in nanograms of the National Institutes of Health Standard (NIH Code F1) (1 ng = 23 ± 3 μIU of WHO 71/222).

Testosterone-3-tyrosine-methyl-ester, LH and prolactin, labelled with $I^{125}$ with the method of chloramine T (Greenwood *et al.*, 1963), 2,4,6,7-[$^3$H]-17β-oestradiol, and the respective antibodies were supplied by Biodata (Milan, Italy).

The coefficients of intra- and inter-assay variation were respectively 9.5% and 18.3% for testosterone, 8% and 15% for oestradiol, 5.5% and 7% for LH, and 5.86% and 9% for prolactin. Comparisons of hormone levels between cancer and control groups and between their subgroups "time since mastectomy" and "years since menopause" were performed by Student's *t*-test, after conversion of data to a logarithmic scale. The correlation between the 4 hormones studied in the control group and in the cancer group and the correlation of each hormone with age at menopause and years since menopause were analyzed by determining the linear coefficient of correlation with the corresponding *P* values. Two-tailed *P* values were considered for all the statistical analyses.

### Results

Mean age, mean age at menopause, mean time since menopause to hormonal examination, and mean age at marriage did not differ significantly between normal controls and breast cancer patients. Mean age at first pregnancy was signficiantly lower in the control group ($P<0.01$) and mean number of full-term pregnancies was significantly lower in the breast cancer group ($P<0.02$) (Table I). Within the breast cancer group, the subgroups 2–12 mo since mastectomy and >2 y since mastectomy did not differ significantly for age, age at menopause and years since menopause.

*Testosterone*

The mean value in the breast cancer group was $0.554 \pm 0.2\ \text{ng ml}^{-1}$ and in normal controls $0.318 \pm 0.11\ \text{ng ml}^{-1}$ ($P<0.001$) (Table II). The distribution was shifted to higher values in the patient group, where we found values exceeding $0.58\ \text{ng ml}^{-1}$ (upper limit for normal controls) in 14 patients.

*Time since mastectomy* The mean testosterone value in the 19 patients examined within 12 mo of mastectomy was higher than in 9 patients examined

**Table I** Age, age at menopause, years since menopause to hormonal examination, age at marriage, age at first term pregnancy and number of full-term pregnancies in normal postmenopausal controls and in postmenopausal breast cancer patients (ranges and mean ±s.d.)

| | *Normal controls* | | | *Breast cancer patients* | | | |
|---|---|---|---|---|---|---|---|
| | *No. cases* | *Range* | *Mean±s.d.* | *No. cases* | *Range* | *Mean±s.d.* | *Significance* |
| Age (y) | 30 | 49–77 | 57.4±6.7 | 28 | 47–77 | 58.5±7.2 | NS* |
| Age at menopause (y) | 30 | 42–55 | 49.6±3.9 | 28 | 43–54 | 48.9±4.4 | NS |
| Years since menopause (y) | 30 | 1–30 | 7.8±7.1 | 28 | 1–28 | 9.9±8.0 | NS |
| Age at marriage (y) | 24 | 18–30 | 23.5±3.1 | 25 | 21–36 | 27.1±4.5 | NS |
| Age at first pregnancy (y) | 24 | 20–32 | 25.1±3.5 | 25 | 23–41 | 29.3±5.2 | $P<0.01$ |
| Number of pregnancies | 24 | 1–6 | 2.6±1.5 | 25 | 1–3 | 1.7±0.8 | $P<0.02$ |

*NS, not significant.

**Table II** Ranges and mean values of testosterone, 17β-oestradiol, luteinising hormone (LH), and prolactin in postmenopausal normal controls and in postmenopausal breast cancer patients

| | *Normal controls* | | | *Breast cancer patients* | | | |
|---|---|---|---|---|---|---|---|
| | *No. cases* | *Range* | *Mean±s.d.* | *No. cases* | *Range* | *Mean±s.d.* | *Significance* |
| Testosterone (ng ml$^{-1}$) | 30 | 0.12– 0.58 | 0.31± 0.11 | 28 | 0.18– 0.99 | 0.55± 0.2 | $P<0.001$ |
| Oestradiol (pg ml$^{-1}$) | 30 | 15.50– 95.50 | 26.45±15.03 | 24 | 15.50– 62.00 | 24.27±10.38 | NS* |
| LH (mIU ml$^{-1}$) | 30 | 35.20–192.50 | 94.73±37.93 | 28 | 28.50–210.00 | 102.82±42.07 | NS |
| Prolactin (ng ml$^{-1}$) | 30 | 3.75– 26.50 | 7.79± 4.78 | 28 | 4.60– 17.50 | 8.94± 3.27 | NS |

*NS, not significant.

years after mastectomy, but the difference was not statistically significant (Table III). In both these subgroups the mean testosterone value was significantly higher than in the control group ($P<0.001$ and $P<0.02$, respectively).

*Years since menopause* In all 3 subgroups of breast cancer patients, mean testosterone levels were higher than in those of normal controls. The difference was statistically significant in the subgroups 1–5 ($P<0.05$) and >10 y since menopause ($P<0.001$) (Table IV). Within the breast cancer group, mean testosterone level significantly increased in the subgroup >10 y since menopause ($P<0.001$). On the other hand, within the control group, a decrease in mean testosterone level was found in late postmenopausal controls (>10 y since menopause), but it was not statistically significant (Table IV).

### *17β-Oestradiol*

The mean value in the breast cancer group did not differ significantly from that of the control group (Table II).

*Time since mastectomy* No statistical difference was found in the mean oestradiol levels between 17 patients examined close to mastectomy and 7 patients examined years after surgery, or between each subgroup and the control group (Table III).

*Years since menopause* No significant difference was found between normal controls and breast cancer patients in any of the 3 subgroups, and no significant difference was found between the 3 subgroups in the control group or in the breast cancer group (Table IV).

### *Luteinising hormone (LH)*

There was a wide range of variation in both breast cancer patients and normal controls, and no significant difference between them (Table II).

*Time since mastectomy* The mean value in the 9 patients examined years after mastectomy was higher than in the 19 patients examined 2–12 mo after surgery, but the difference was not statistically significant ($t=2.0$). No statistical difference was found between each subgroup and the control group (Table III).

**Table III** Ranges and mean values of testosterone, 17β-oestradiol, luteinising hormone (LH), and prolactin in 19 postmenopausal breast cancer patients examined within 12 months of mastectomy (Group 1) and in 9 patients examined more than 2 years after surgery (Group 2)

| | *Group 1* | | | *Group 2* | | | |
|---|---|---|---|---|---|---|---|
| | *No. cases* | *Range* | *Mean ± s.d.* | *No. cases* | *Range* | *Mean ± s.d.* | *Significance* |
| Testosterone ($ng\,ml^{-1}$) | 19 | 0.19– 0.99 | 0.58 ± 0.2* | 9 | 0.18– 0.76 | 0.48 ± 0.2† | NS‡ |
| Oestradiol ($pg\,ml^{-1}$) | 17 | 15.50– 40.00 | 23.67 ± 7.3§ | 7 | 15.50– 62.00 | 25.70 ± 16.3§ | NS |
| LH ($mIU\,ml^{-1}$) | 19 | 28.50–210.00 | 95.09 ± 43.6§ | 9 | 81.00–200.00 | 119.15 ± 35.3§ | NS |
| Prolactin ($ng\,ml^{-1}$) | 19 | 4.60– 17.50 | 9.04 ± 3.5§ | 9 | 5.80– 16.00 | 8.80 ± 3.0§ | NS |

*Statistical significance vs. normal controls, $P < 0.001$.
†Statistical significance vs. normal controls, $P < 0.02$.
‡NS, not significant.
§Statistical significane vs. normal controls, not significant.

**Table IV** Mean values ± s.d. of testosterone, 17β-oestradiol, luteinising hormone (LH), and prolactin in normal postmenopausal controls and in postmenopausal breast cancer patients divided into three subgroups according to years since menopause

| | *Normal controls* | | *Breast cancer patients* | | |
|---|---|---|---|---|---|
| *Years since menopause* | *No. cases* | *Mean ± s.d.* | *No. cases* | *Mean ± s.d.* | *Significance* |
| 1–5 | | | | | |
| Testosterone ($ng\,ml^{-1}$) | 12 | 0.33 ± 0.08 | 10 | 0.48 ± 0.18* | $P < 0.05$ |
| Oestradiol ($pg\,ml^{-1}$) | 12 | 30.37 ± 22.60 | 10 | 23.50 ± 6.40 | NS† |
| LH ($mIU\,ml^{-1}$) | 12 | 103.00 ± 46.90 | 10 | 110.90 ± 34.70 | NS |
| Prolactin ($ng\,ml^{-1}$) | 12 | 8.90 ± 6.80 | 10 | 8.70 ± 2.40 | NS |
| 6–10 | | | | | |
| Testosterone ($ng\,ml^{-1}$) | 11 | 0.34 ± 0.14 | 8 | 0.48 ± 0.19* | NS |
| Oestradiol ($pg\,ml^{-1}$) | 11 | 24.20 ± 6.40 | 7 | 29.00 ± 17.10 | NS |
| LH ($mIU\,ml^{-1}$) | 11 | 100.90 ± 32.80 | 8 | 111.90 ± 41.80 | NS |
| Prolactin ($ng\,ml^{-1}$) | 11 | 7.90 ± 3.00 | 8 | 8.90 ± 4.00 | NS |
| > 10 | | | | | |
| Testosterone ($ng\,ml^{-1}$) | 7 | 0.27 ± 0.10 | 10 | 0.68 ± 0.18 | $P < 0.001$ |
| Oestradiol ($pg\,ml^{-1}$) | 7 | 23.30 ± 5.40 | 7 | 20.60 ± 3.90 | NS |
| LH ($mIU\,ml^{-1}$) | 7 | 70.90 ± 16.20 | 10 | 87.40 ± 34.10 | NS |
| Prolactin ($ng\,ml^{-1}$) | 7 | 5.70 ± 1.60 | 10 | 9.20 ± 3.60 | $P < 0.02$ |

*Statistical significance between 1–5 and > 10 y since menopause subgroups and between 6–10 and > 10 y since menopause subgroups, within breast cancer group, $P < 0.001$.
†NS, not significant.

*Years since menopause* No difference was found in the 3 subgroups between normal controls and breast cancer patients, or between the 3 subgroups within the breast cancer group or within the control group (Table IV).

### *Prolactin*

The distribution was slightly but not significantly shifted to higher values in the patient group (Table II).

*Time since mastectomy* No difference was found in the mean prolactin levels between the 19 patients examined 2–12 mo after mastectomy and the 9 patients examined years after surgery, or between each subgroup and the control group (Table III).

*Years since menopause* A statistically significant difference was found between controls and patients in late postmenopause (> 10 y since menopause) ($P < 0.02$) but not in the other subgroups. No significant difference was found between the 3

subgroups, either within the control group or within the breast cancer group (Table IV).

*Correlations*

No correlation was found between the 4 hormones studied, either in the control group or in the breast cancer group, considering the overall groups or dividing them into subgroups according to years since menopause or, for the patient group, according to time since mastectomy. A statistically significant correlation was found between testosterone and years since menopause ($r = 0.533$, $P < 0.01$) in breast cancer patients but not in normal controls. Years since menopause were not correlated with oestradiol, LH or prolactin levels in either group. No correlation was found between any of the 4 hormones studied and age at menopause in the control group, the breast cancer group, or their subgroups.

### Discussion

The present results show that mean values of serum oestradiol, LH and prolactin are similar in postmenopausal women with breast cancer and normal controls. These findings are in agreement with most data from the literature for oestradiol (Bird *et al.*, 1981; Borkowski *et al.*, 1977; England *et al.*, 1977; Jones *et al.*, 1977; Malarkey *et al.*, 1977; McFadyen *et al.*, 1976), LH (Bird *et al.*, 1981; Malarkey *et al.*, 1977; McFadyen *et al.*, 1977) and prolactin levels (Franks *et al.*, 1974; Jones *et al.*, 1977; Kwa *et al.*, 1974; Mittra *et al.*, 1974; Sheth *et al.*, 1975).

In our series, testosterone levels in breast cancer patients were significantly higher than in normal controls, considering either the overall groups or dividing them into subgroups according to years since menopause or, for the patient group, according to time since mastectomy.

These data confirm the findings of our previous papers (Grattarola *et al.*, 1974, 1975), in which urinary testosterone values higher than normal were found in postmenopausal breast cancer patients. Reports of blood testosterone levels in these patients are few and conflicting (Adami *et al.*, 1979; Bird *et al.*, 1981; Borkowski *et al.*, 1977; Horn & Gordan, 1974; Malarkey *et al.*, 1977; McFadyen *et al.*, 1976). In his review of plasma hormone concentration in human breast cancer, Zumoff (1978) pointed out that "in most studies the groups were heterogeneous, complicated by other diseases and/or medications, and poorly or not at all characterized." Our patients were rigidly selected according to predetermined criteria. In the control group, testosterone values were in agreement with normal values in most other reports (Adami *et al.*, 1979; Bird *et al.*, 1981; Malarkey *et al.*, 1977; Vermeulen, 1976).

Our data have been obtained on the basis of a single "spot" sample, which has been criticized (Zumoff, 1978). Diurnal variations of testosterone in postmenopausal women were shown by Vermeulen (1976), with highest levels at 8 a.m. and a significant decrease at 8 p.m., but the differences between 8 a.m. and 12 a.m. were very small. Time of sampling in our series, both for controls and breast cancer patients, was between 9 a.m. and 11 a.m. We feel it is unlikely that difference in sampling time can account for the highly significant difference in testosterone values between the control group and the breast cancer group, where 50% of the patients had testosterone levels higher than the highest testosterone value in the controls. Furthermore, the correlation of years since menopause with testosterone but not with the other hormones is highly suggestive of some abnormality just in testosterone production and metabolism rather than for some casual event.

Adipose tissue aromatizes androgens to oestrogens (Nimrod & Ryan, 1975) and body weight and the percentage of conversion of androstenedione to oestrone are highly correlated in women (Siiteri & MacDonald, 1973). A highly significant correlation was found between body weight and circulating oestrogens in normal postmenopausal subjects and in patients with endometrial adenocarcinoma (Frumar *et al.*, 1980; Judd *et al.*, 1980), but no correlation was present between body weight and testosterone. Body weight was recorded in about half of our patients, and it did not differ from that of the controls.

There is evidence that postmenopausal ovaries continue to secrete significant amounts of androgenic hormones (Judd *et al.*, 1974*a*, 1974*b*; Schenker *et al.*, 1979; Vermeulen, 1976), whereas oestrogens after the menopause are derived almost completely from the peripheral conversion of androgens (Grodin *et al.*, 1973). Androgens are known to be synthesized in the interstitial tissue of the ovary (Rice & Savard, 1966), and a high incidence of hyperplastic ovarian interstitial tissue has been described in breast cancer patients (Smith & Smith, 1953; Sommers, 1955; Sommers & Teloh, 1952). A previous study from our laboratory (Grattarola, 1976) showed ovarian interstitial hyperplasia and elevated urinary testosterone excretion in a large number of pre- and post-menopausal breast cancer patients submitted to ovariectomy.

We can surmise that ovarian interstitial hyperplasia is more frequent in breast cancer patients than in normal controls and that the hyperplastic tissue, under stimulation of elevated gonadotropin levels, continues to produce larger and larger amounts of testosterone with the years since menopause. On the other hand, hormonal production in normal postmenopausal ovaries is steady or slightly decreased with years since menopause.

In conclusion, these data confirm our previous findings of increased urinary testosterone in breast cancer patients (Grattarola *et al.*, 1974, 1975), and further support the hypothesis that hyperandrogenism may play a role in the development of breast cancer.

Although our data cannot answer the question whether the abnormality in testosterone levels is simply a result of mastectomy itself or whether it was present preoperatively and indicates a true metabolic difference between those women destined to develop breast cancer and those who are not, we surmise that the latter hypothesis is more probable. In fact, increased urinary excretion of testosterone and androstanediol was reported (Grattarola, 1978) in patients with hyperplastic alterations of breast epithelium, which is a risk factor for breast cancer, and we recently found urinary testosterone and androstanediol excretion higher than normal in 39% of patients with hyperplasia and in 61% of patients with cancer of the breast, examined before mastectomy (Secreto *et al.*, 1982).

### References

ADAMI, H.-O., JOHANSSON, E.D.B., VEGELIUS, J. & FICTOR, A. (1979). Serum concentrations of estrone, androstenedione, testosterone and sex-hormone-binding globulin in postmenopausal women with breast cancer and in age-matched controls. *Upsala J. Med. Sci.*, **84**, 259.

BIRD, C.E., COOK, S., OWEN, S., STERNS, E.E. & CLARK, A.F. (1981). Plasma concentrations of C-19 steroids, estrogens, FSH, LH and prolactin in post-menopausal women with and without breast cancer. *Oncology*, **38**, 365.

BORKOWSKI, A., L'HERMITE, M., DOR, P. & 4 others. (1977). Steroid sex hormones and prolactin in postmenopausal women with generalized mammary carcinoma during prolonged dexamethasone treatment. *J. Endocrinol.*, **73**, 235.

COLLINS, W.P., MANSFIELD, M.D., ALLADINA, N.S. & SOMMERVILLE, I.F. (1972). Radioimmunoassay of plasma testosterone. *J. Steroid Biochem.*, **3**, 333.

DESBUQUOIS, B. & AURBACH, G.D. (1971). Use of polyethylene glycol to separate free and antibody-bound peptide hormones in radioimmuno-assays. *J. Clin. Endocrinol. Metab.*, **33**, 732.

ENGLAND, P.C., SKINNER, L.G., COTTRELL, K.M. & SELLWOOD, R.A. (1975). Sex hormones in breast disease. *Br. J. Surg.*, **62**, 806.

FRANKS, S., RALPHS, D.N.L., SEAGROATT, V. & JACOBS, H.S. (1974). Prolactin concentrations in patients with breast cancer. *Br. Med. J.*, **9**, 320.

FRUMAR, A.M., MELDRUM, D.R., GEOLA, F. & 4 others. '1980). Relationship of fasting urinary calcium to circulating estrogen and body weight in postmenopausal women. *J. Clin. Endocrinol. Metab.*, **50**, 70.

GRATTAROLA, R. (1976). Ovariectomy alone or in combination with dexamethasone in patients with advanced breast cancer and high levels of testosterone excretion. *J. Natl Cancer Inst.*, **56**, 11.

GRATTAROLA, R. (1978). Anovulation and increased androgenic activity as breast cancer risk in women with fibrocystic disease of the breast. *Cancer Res.*, **38**, 3051.

GRATTAROLA, R., SECRETO, G., RECCHIONE, C. & CASTELLINI, W. (1974). Androgens in breast cancer. II. Endometrial adenocarcinoma and breast cancer in married postmenopausal women. *Am. J. Obstet. Gynecol.*, **118**, 173.

GRATTAROLA, R., SECRETO, G. & RECCHIONE, C. (1975). Androgens in breast cancer. III. Breast cancer recurrences years after mastectomy and increased androgenic activity. *Am. J. Obstet. Gynecol.*, **121**, 169.

GREENWOOD, F.C., HUNTER, W.M. & GLOVER, J.S. (1963). The preparation of $^{131}$I-labeled growth hormone of high specific radioactivity. *Biochem. J.*, **89**, 114.

GRODIN, J.H., SIITERI, P.K. & MacDONALD, P.C. (1973). Source of estrogen production in postmenopausal women. *J. Clin. Endocrinol. Metab.*, **36**, 207.

HORN, H. & GORDON, G.S. (1974). Plasma testosterone in advanced breast cancer. *Oncology*, **30**, 147.

ISMAIL, A.A.A., NISWENDER, G.D. & MIDGLEY, A.R. JR. (1972). Radioimmunoassay of testosterone without chromatography. *J. Clin. Endocrinol.*, **34**, 177.

JONES, M.K., RAMSAY, I.D., BOOTH, M. & COLLINS, W.P. (1977). Hormone concentrations in postmenopausal patients with breast cancer. *Clin. Oncol.*, **3**, 177.

JUDD, H.L., DAVIDSON, B.J., FRUMAR, A.M., SHAMONKI, I.M., LAGASSE, L.D. & BALLON, S.C. (1980). Serum androgens and estrogens in postmenopausal women with and without endometrial cancer. *Am. J. Obstet. Gynecol.*, **136**, 859.

JUDD, H.L., JUDD, G.E., LUCAS, W.E. & YEN, S.S.C. (1974*a*). Endocrine function of the postmenopausal ovary: Concentration of androgens and estrogens in ovarian and peripheral vein blood. *J. Clin. Endocrinol. Metab.*, **39**, 1020.

JUDD, H.L., LUCAS, W.E. & YEN, S.S.C. (1974*b*). Effect of oophorectomy on circulating testosterone and androstenedione levels in patients with endometrial cancer. *Am. J. Obstet. Gynecol.*, **118**, 793.

KWA, H.G., ENGELSMAN, E., DE JONG-BAKKER, M. & CLETON, F.J. (1974). Plasma-prolactin in human breast cancer. *Lancet*, **i**, 433.

MALARKEY, W.B., SCHROEDER, L.L., STEVENS, V.C., JAMES, A.G. & LANESE, R.R. (1977). Twenty-four-hour preoperative endocrine profiles in women with benign and malignant breast disease. *Cancer Res.*, **37**, 4655.

McFADYEN, I.J., PRESCOTT, R.J., GROOM, G.V. & 4 others. (1976). Circulating hormone concentrations in women with breast cancer. *Lancet*, **i**, 1100.

MIDGLEY, A.R. JR. (1966). Radioimmunoassay: A method for human chorionic gonadotropin and human luteinizing hormone. *Endocrinology*, **79**, 10.

MITTRA, I., HAYWARD, J.L. & McNEILLY, A.S. (1974). Hypothalamic-pituitary-prolactin axis in breast cancer. *Lancet*, **i**, 889.

NAGASAWA, H. (1979). Prolactin and human breast cancer: A review. *Eur. J. Cancer*, **15**, 267.

NIMROD, A. & RYAN, K.J. (1975). Aromatization of androgens by human abdominal and breast fat tissue. *J. Clin. Endocrinol. Metab.*, **40**, 367.

NISKER, J.A. & SIITERI, P.K. (1981). Estrogens and breast cancer. *Clin. Obstet. Gynecol.*, **24**, 301.

RICCI, G. & SACCONI, G. (1976). Radioimmunoassay of 17$\beta$-estradiol. *Biodata Bull.*, **1**, 9.

RICE, B.F. & SAVARD, K. (1966). Steroid hormone formation in the human ovary. IV. Ovarian stromal compartment: formation of radioactive steroids from acetate-1-$C^{14}$ and action of gonadotropins. *J. Clin. Endocrinol.*, **26**, 593.

SCHENKER, J.G., WEINSTEIN, D. & OKON, E. (1979). Estradiol and testosterone levels in the peripheral and ovarian circulations in patients with endometrial cancer. *Cancer*, **44**, 1809.

SECRETO, G., FARISELLI, G., BANDIERAMONTE, G., RECCHIONE, C., DATI, V. & DI PIETRO, S. (1982). Androgen excretion in women with family history of breast cancer or with epithelial hyperplasia or cancer of the breast. *Eur. J. Cancer*, (in press).

SHETH, N.A., RANADIVE, K.J., SURAIYA, J.N. & SHETH, A.R. (1975). Circulating levels of prolactin in human breast cancer. *Br. J. Cancer*, **32**, 160.

SIITERI, P.K. & MACDONALD, P.C. (1973). Role of extraglandular estrogen in human endocrinology. In *Handbook of Physiology, Section 7, Endocrinology*, Vol. 2, (Ed. Greef). Washington, D.C.: American Physiological Society. p. 615.

SINHA, Y.N., SELBY, F.W., LEWIS, U.J. & VANDERLAAN, W.P. (1973). A homologous radioimmunoassay for human prolaction. *J. Clin. Endocrinol. Metab.*, **36**, 509.

SMITH, G.V. & SMITH, O.W. (1953). Carcinoma of the breast: Results, evaluation of X-radiation and relation to age and surgical castration to length of survival. *Surg. Gynecol. Obstet.*, **97**, 508.

SOMMERS, S.C. (1955). Endocrine abnormality in women with breast cancer. *Lab. Invest.*, **4**, 160.

SOMMERS, S.C. & TELOH, H.A. (1952). Ovarian stromal hyperplasia in breast cancer. *Arch. Pathol.*, **53**, 160.

VERMEULEN, A. (1976). The hormonal activity of the postmenopausal ovary. *J. Clin. Endocrinol. Metab.*, **42**, 247.

ZUMOFF, B. (1978). Plasma hormone concentrations in human breast and prostate cancer. In *Endocrine Control in Neoplasia*, (Eds. Sharma & Criss). New York: Raven Press. p. 349.

ZUMOFF, B. (1981). The role of endogenous estrogen excess in human breast cancer (review). *Anticancer Res.*, **1**, 39.


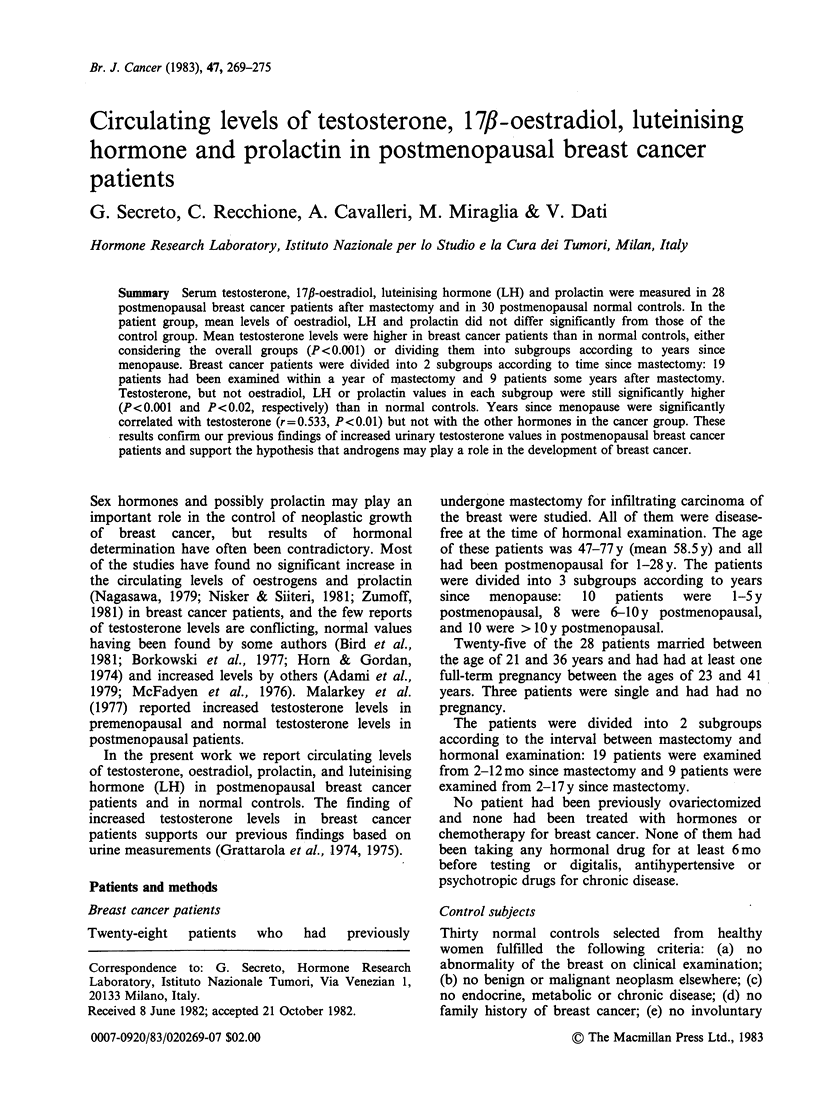

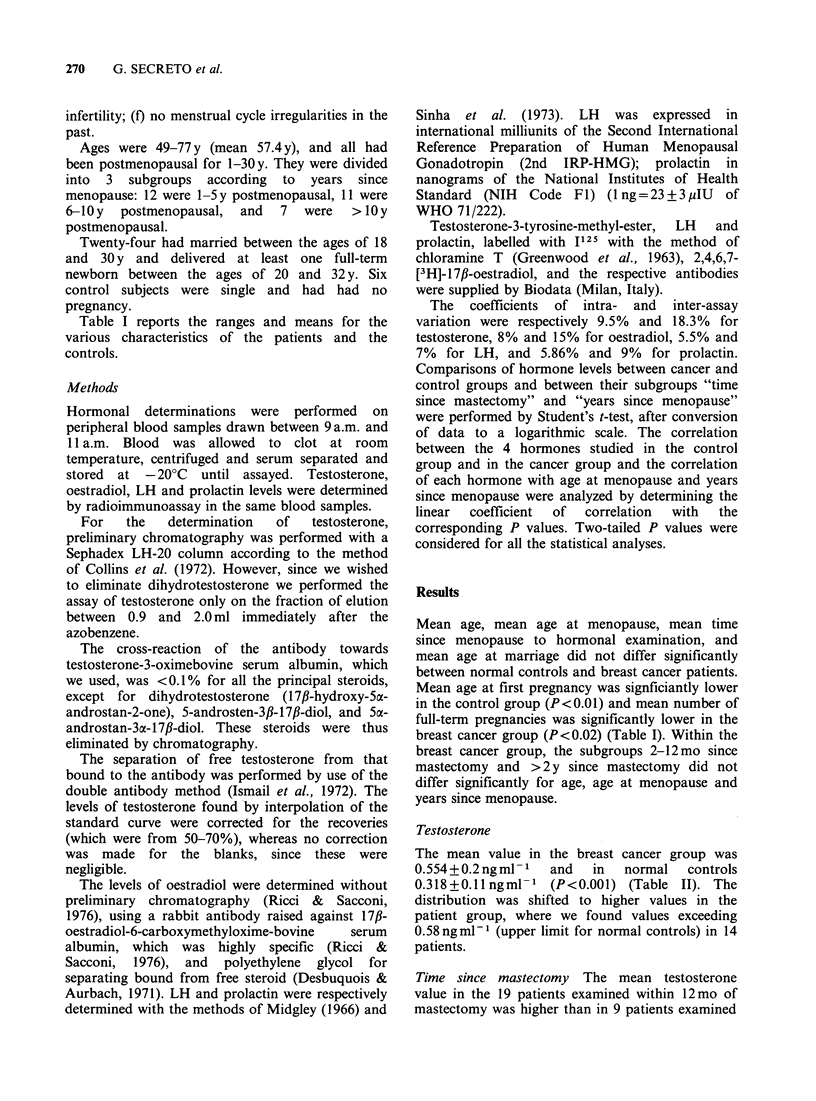

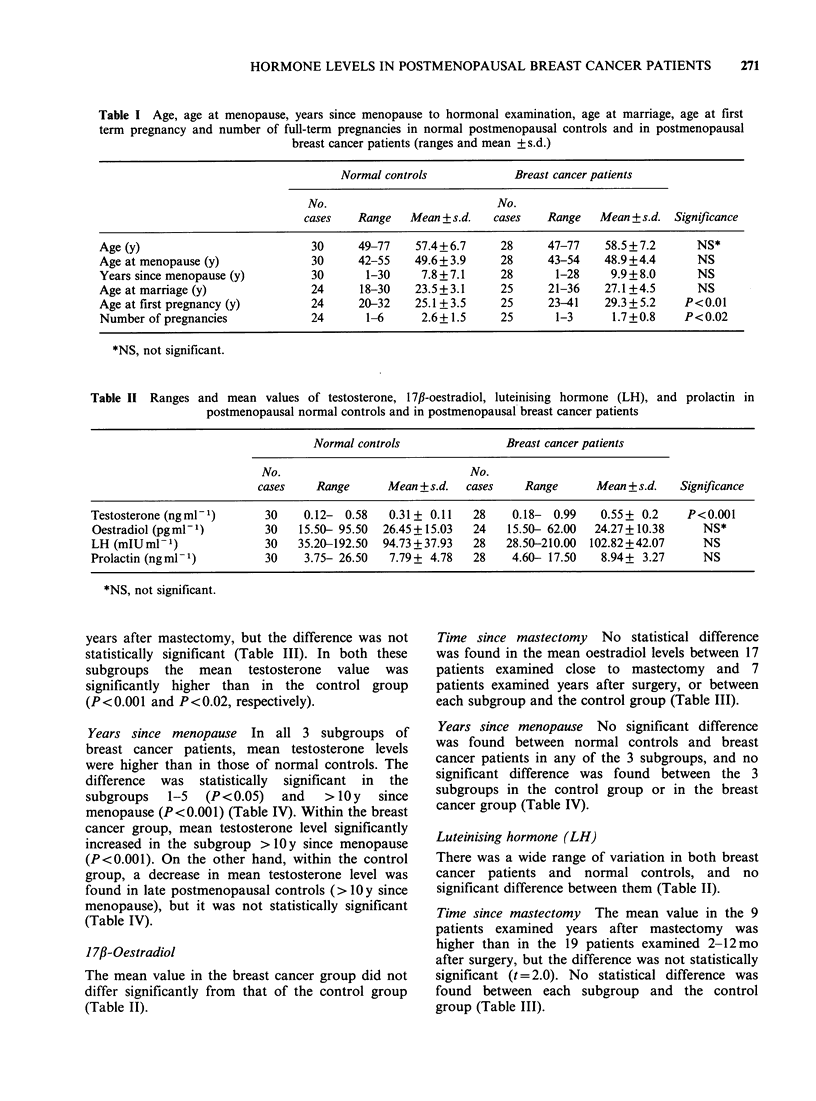

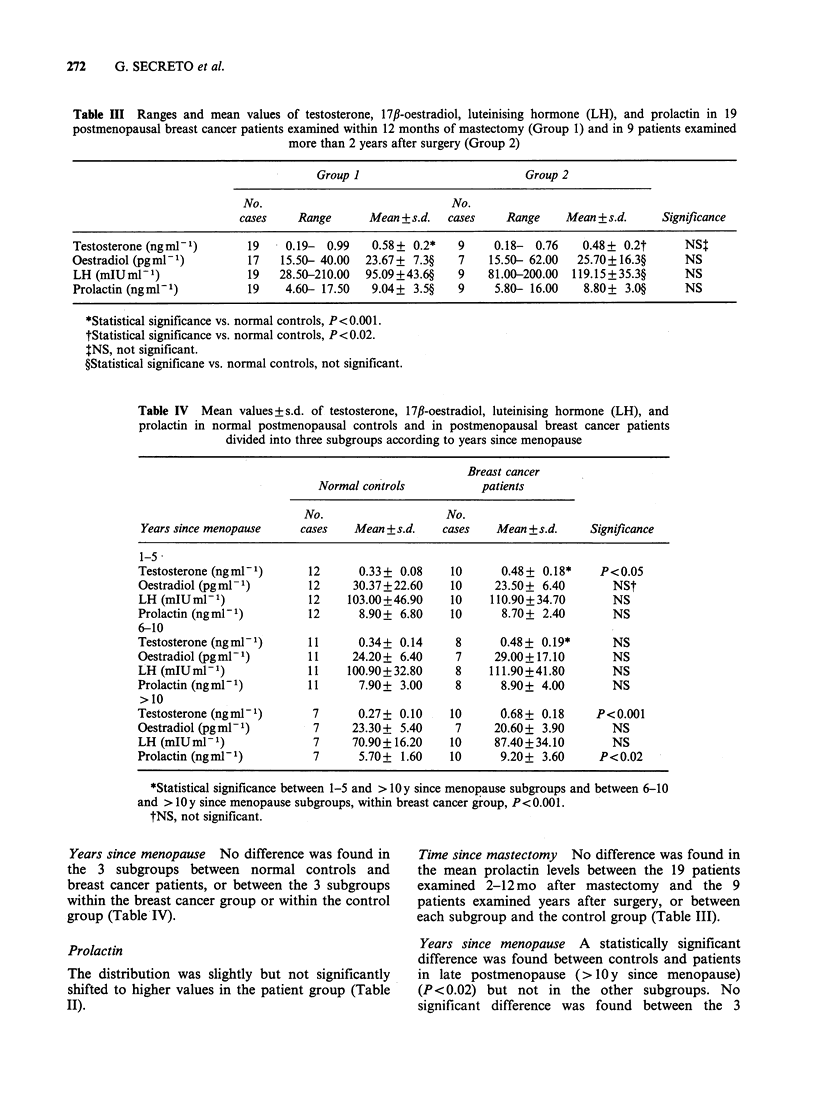

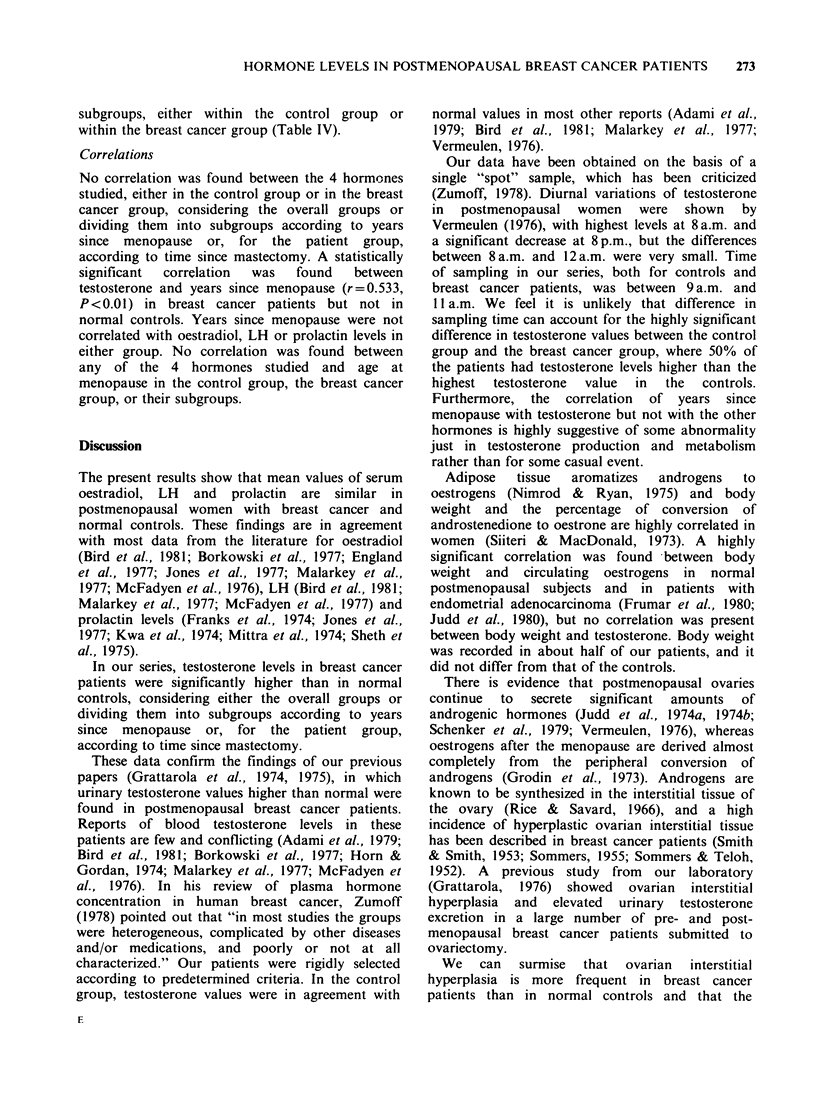

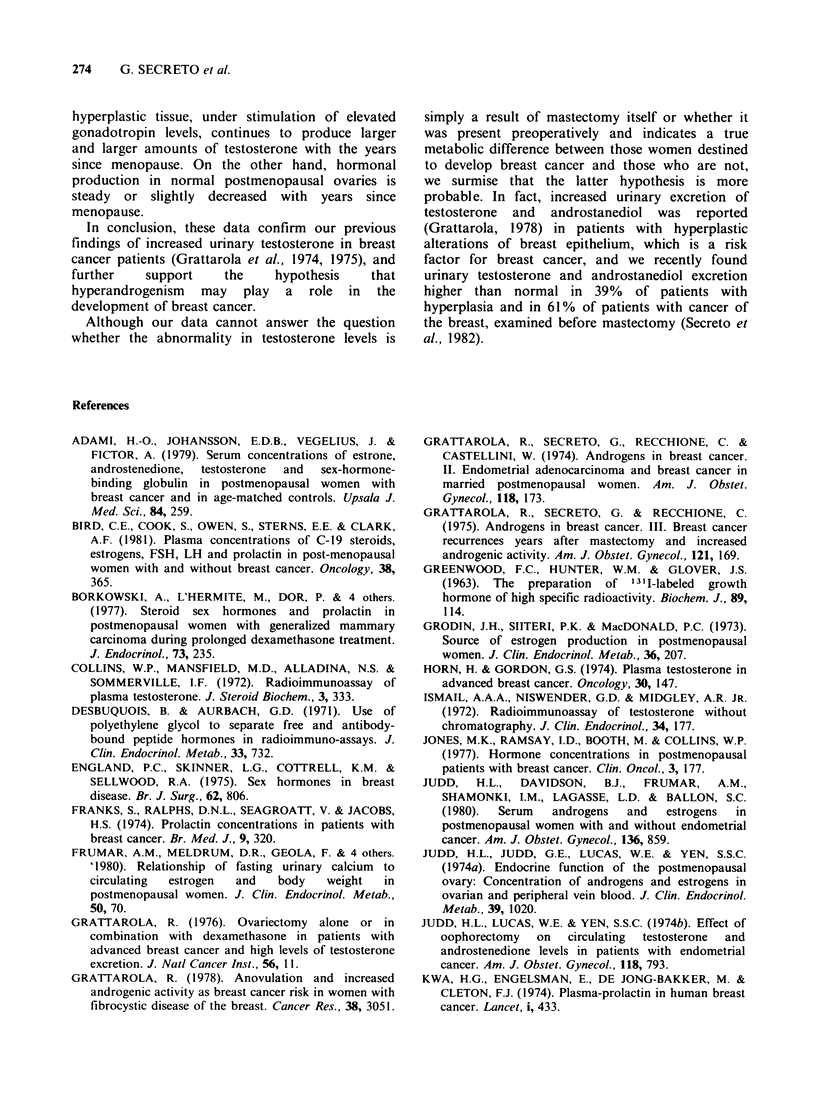

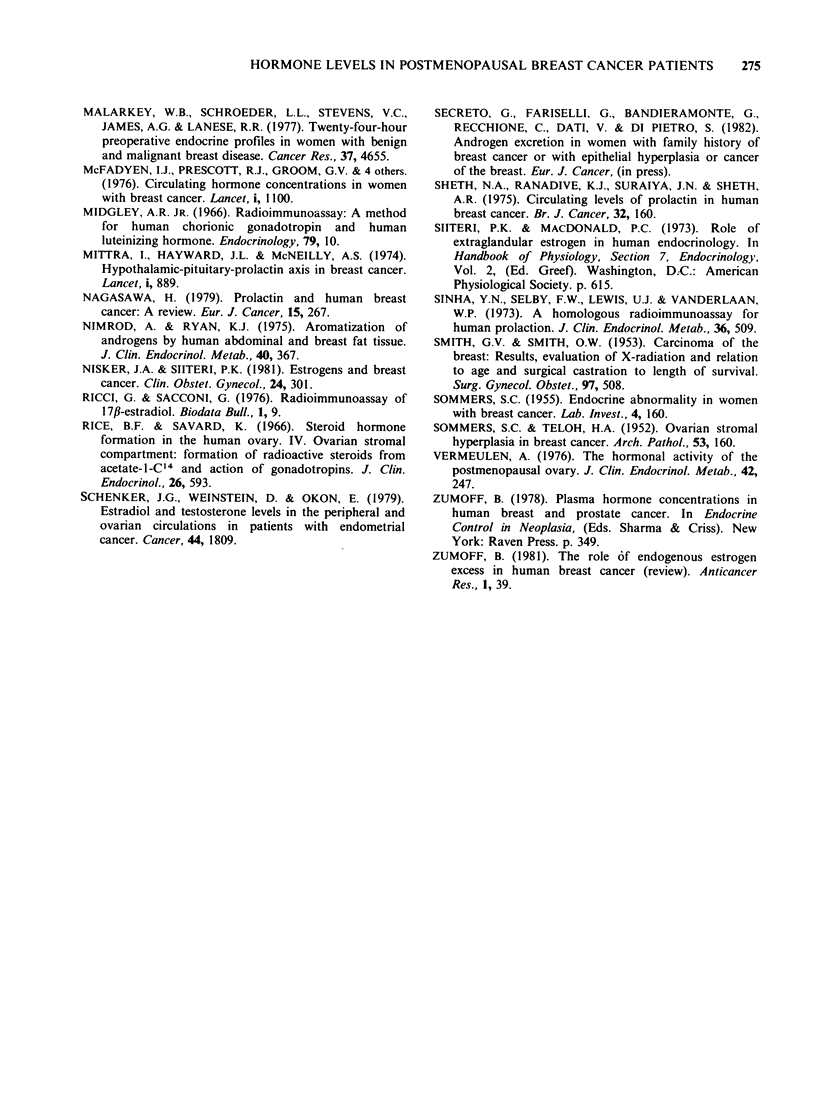


## References

[OCR_00565] Adami H. O., Johansson E. D., Vegelius J., Victor A. (1979). Serum concentrations of estrone, androstenedione, testosterone and sex-hormone-binding globulin in postmenopausal women with breast cancer and in age-matched controls.. Ups J Med Sci.

[OCR_00573] Bird C. E., Cook S., Owen S., Sterns E. E., Clark A. F. (1981). Plasma concentrations of C-19 steroids, estrogens, FSH, LH and prolactin in post-menopausal women with an without breast cancer.. Oncology.

[OCR_00580] Borkowski A., L'hermite M., Dor P., Longeval E., Rozencweig M., Muquardt C., Van Cauter E. (1977). Steroid sex hormones and prolactin in postmenopausal women with generalized mammary carcinoma during prolonged dexamethasone treatment.. J Endocrinol.

[OCR_00587] Collins W. P., Mansfield M. D., Alladina N. S., Sommerville I. F. (1972). Radioimmunoassay of plasma testosterone.. J Steroid Biochem.

[OCR_00592] Desbuquois B., Aurbach G. D. (1971). Use of polyethylene glycol to separate free and antibody-bound peptide hormones in radioimmunoassays.. J Clin Endocrinol Metab.

[OCR_00598] England P. C., Skinner L. G., Cottrell K. M., Sellwood R. A. (1975). Sex hormones in breast disease.. Br J Surg.

[OCR_00603] Franks S., Ralphs D. N., Seagroatt V., Jacobs H. S. (1974). Prolactin concentrations in patients with breast cancer.. Br Med J.

[OCR_00639] GREENWOOD F. C., HUNTER W. M., GLOVER J. S. (1963). THE PREPARATION OF I-131-LABELLED HUMAN GROWTH HORMONE OF HIGH SPECIFIC RADIOACTIVITY.. Biochem J.

[OCR_00615] Grattarola R. (1976). Ovariectomy alone or in combination with dexamethasone in patients with advanced breast cancer and high levels of testosterone excretion.. J Natl Cancer Inst.

[OCR_00633] Grattarola R., Secreto G., Recchione C. (1975). Androgens in breast cancer. III. Breast cancer recurrences years after mastectomy and increased androgenic activity.. Am J Obstet Gynecol.

[OCR_00626] Grattarola R., Secreto G., Recchione C., Castellini W. (1974). Androgens in breast cancer. II. Endometrial adenocarcinoma and breast cancer in married postmenopausal women.. Am J Obstet Gynecol.

[OCR_00621] Gratterola R. (1978). Anovulation and increased androgenic activity as breast cancer risk in women with fibrocystic disease of the breast.. Cancer Res.

[OCR_00645] Grodin J. M., Siiteri P. K., MacDonald P. C. (1973). Source of estrogen production in postmenopausal women.. J Clin Endocrinol Metab.

[OCR_00650] Horn H., Gordan G. S. (1974). Plasma testosterone in advanced breast cancer.. Oncology.

[OCR_00654] Ismail A. A., Niswender G. D., Midgley A. R. (1972). Radioimmunoassay of testosterone without chromatography.. J Clin Endocrinol Metab.

[OCR_00659] Jones M. K., Ramsay I. D., Booth M., Collins W. P. (1977). Hormone concentrations in postmenopausal patients with breast cancer.. Clin Oncol.

[OCR_00664] Judd H. L., Davidson B. J., Frumar A. M., Shamonki I. M., Lagasse L. D., Ballon S. C. (1980). Serum androgens and estrogens in postmenopausal women with and without endometrial cancer.. Am J Obstet Gynecol.

[OCR_00671] Judd H. L., Judd G. E., Lucas W. E., Yen S. S. (1974). Endocrine function of the postmenopausal ovary: concentration of androgens and estrogens in ovarian and peripheral vein blood.. J Clin Endocrinol Metab.

[OCR_00678] Judd H. L., Lucas W. E., Yen S. S. (1974). Effect of oophorectomy on circulating testosterone and androstenedione levels in patients with endometrial cancer.. Am J Obstet Gynecol.

[OCR_00684] Kwa H. G., De Jong-Bakker M., Engelsman E., Cleton F. J. (1974). Plasma-prolactin in human breast cancer.. Lancet.

[OCR_00691] Malarkey W. B., Schroeder L. L., Stevens V. C., James A. G., Lanese R. R. (1977). Twenty-four-hour preoperative endocrine profiles in women with benign and malignant breast disease.. Cancer Res.

[OCR_00697] McFadyen I. J., Forrest A. P., Prescott R. J., Golder M. P., Groom G. V., Fahmy D. R., Griffiths K. (1976). Circulating hormone concentrations in women with breast cancer.. Lancet.

[OCR_00702] Midgley A. R. (1966). Radioimmunoassay: a method for human chorionic gonadotropin and human luteinizing hormone.. Endocrinology.

[OCR_00707] Mittra I., Hayward J. L., McNeilly A. S. (1974). Hypothalamic-pituitary-prolactin axis in breast cancer.. Lancet.

[OCR_00712] Nagasawa H. (1979). Prolactin and human breast cancer: a review.. Eur J Cancer.

[OCR_00716] Nimrod A., Ryan K. J. (1975). Aromatization of androgens by human abdominal and breast fat tissue.. J Clin Endocrinol Metab.

[OCR_00721] Nisker J. A., Siiteri P. K. (1981). Estrogens and breast cancer.. Clin Obstet Gynecol.

[OCR_00729] Rice B. F., Savard K. (1966). Steroid hormone formation in the human ovary. IV. Ovarian stromal compartment; formation of radioactive steroids from acetate-1-14C and action of gonadotropins.. J Clin Endocrinol Metab.

[OCR_00765] SMITH G. V., SMITH O. W. (1953). Carcinoma of the breast; results, evaluation of X-radiation, and relation of age and surgical castration to length of survival.. Surg Gynecol Obstet.

[OCR_00771] SOMMERS S. C. (1955). Endocrine abnormalities in women with brest cancer.. Lab Invest.

[OCR_00775] SOMMERS S. C., TELOH H. A. (1952). Ovarian stromal hyperplasia in breast cancer.. AMA Arch Pathol.

[OCR_00736] Schenker J. G., Weinstein D., Okon E. (1979). Estradiol and testosterone levels in the peripheral and ovarian circulations in patients with endometrial cancer.. Cancer.

[OCR_00749] Sheth N. A., Ranadive K. J., Suraiya J. N., Sheth A. R. (1975). Circulating levels of prolactin in human breast cancer.. Br J Cancer.

[OCR_00761] Sinha Y. N., Selby F. W., Lewis U. J., VanderLaan W. P. (1973). A homologous radioimmunoassay for human prolactin.. J Clin Endocrinol Metab.

[OCR_00779] Vermeulen A. (1976). The hormonal activity of the postmenopausal ovary.. J Clin Endocrinol Metab.

